# Plasma is ineffective in correcting mildly elevated PT-INR in critically ill children: a retrospective observational study

**DOI:** 10.1186/s40560-014-0064-1

**Published:** 2014-11-29

**Authors:** Esther Paula Soundar, Ronald Besandre, Sarah Kate Hartman, Jun Teruya, Shiu-Ki Rocky Hui

**Affiliations:** Division of Transfusion Medicine and Coagulation, Texas Childrens’ Hospital, 6621 Fannin Street, Suite WB 1100, Houston, TX 77030 USA; Department of Pathology and Immunology, Baylor College of Medicine, One Baylor Plaza, Houston, TX 77030 USA

**Keywords:** Children, Plasma transfusion, Mildly prolonged PT-INR, Coagulopathy, Pediatric intensive care unit, Guidelines

## Abstract

**Background:**

Fresh frozen plasma transfusion is widely utilized in pediatric clinical practice to correct mild coagulopathy. Several studies on adult population have shown that transfusion of plasma cannot effectively correct mild coagulopathy when international normalized ratio (INR) is ≤1.5. Much controversy exists about the generalization of this finding for pediatric populations, especially since pediatric dosages often exceed those in adults. The aim of this study is to determine the prevalence of plasma transfusion with mild coagulopathy (INR ≤ 1.5) and its effectiveness in a pediatric setting.

**Methods:**

In our tertiary referral hospital, we retrospectively reviewed the electronic medical records of all patients who received plasma (April to October 2011) for mildly elevated prothrombin time (PT)-INR levels (≤1.5) and had post-transfusion PT-INR measurements; patients who received intraoperative, ECMO, or plasma exchange-related plasma transfusions were excluded from this study. We abstracted demographic data and pre- and post coagulation test results for the patients included in our study.

**Results:**

Among 468 plasma transfusions administered to 285 patients from April to June 2011, 60 plasma transfusions (12.8%) were given to patients with PT-INR ≤ 1.5 (range 1.3–1.5). Forty-one patients [median age 2.5 years (IQR, 0.14 to 13.75 years), median weight of 16.0 kg (IQR, 8.0 to 69.3 kg)] who received 41 single plasma transfusions [median dose 11 mL/Kg (IQR, 6–15)] had post-transfusion PT-INR measurements and were included in our study. There was no significant difference in their PT-INR values (*p* = 0.34) pre- and post-transfusion. Of our study, only 15.4% patients showed post-transfusion normalization [median change in PT-INR 0.15 (IQR, 0.1–0.2)] and were not different from the remaining 85% in age, plasma dose, and bleeding status.

**Conclusions:**

The prevalence of plasma transfusion for correction of mildly elevated PT-INR levels in critically ill children is high (12.8%). Plasma transfusion showed no significant effect in correcting minor prolongation of PT-INR in pediatric patients regardless of age, volume of plasma transfused per kilogram (dosage), or bleeding status.

## Background

Fresh frozen plasma (FFP) constitutes a ready supply of plasma proteins, most notably the coagulation-related factors. Its utilization resides chiefly in replenishing coagulation factors in any clinical setting with ongoing or suspected coagulopathy. As can be expected from a product with broad indications, plasma usage in the United States is expanding and evolving along with medical and surgical support capabilities. In particular, studies of plasma use in pediatric intensive care units (PICUs) demonstrate more frequent use of plasma to support volume expansion, massive hemorrhage, liver disease, disseminated intravascular coagulation, and correction of prolonged clotting tests, among other clinical indications [1]. Appropriate use of plasma is indicated in massive hemorrhage, liver disease, and disseminated intravascular coagulopathy (DIC) with bleeding in pediatric intensive care settings [1,2]. Its role as an effective prophylactic agent, however, has been disputed when used prior to invasive procedures such as liver biopsy and central line placement, with no correlation found between bleeding risk and pre-procedure coagulation test results [3,4]. In addition, plasma transfusion carries its own host of complications, including disease transmission, allergic reactions, alloimmunization, volume overload, thrombosis, multiple organ failure, and transfusion-related acute lung injury (TRALI) [5-10]. The majority of studies evaluating outcome of plasma transfusion in specific clinical scenarios, particularly in neonates, have prompted the recent publication of guidelines to curtail plasma transfusion for mild coagulation abnormalities in children [11]. According to recent transfusion data issued by hospitals in the United States, the United Kingdom, and Canada, prescribing plasma inappropriately is a common practice, despite published guidelines [12,13].

The current plasma transfusion practice for the purpose of correcting mildly prolonged coagulation tests is of interest to our institution, the largest pediatric hospital in the United States. Published studies on adult plasma transfusion have shown minimal to no effect when used either prophylactically or as a therapeutic agent for bleeding when the international normalized ratio (INR) is ≤1.5 [14-16]. Puetz et al. recently demonstrated pediatric United States hospitals to have a high prevalence of plasma transfusion in critically ill children but did not evaluate the indications for transfusion [17]. We set out to evaluate the local transfusion practice with respect to plasma by: 1) determining the frequency of plasma transfusions and 2) assessing the significance of improvements in coagulation tests [prothrombin time-international normalized ratio (PT-INR)] in the context of the clinical scenario via the electronic medical record. In the United States, most transfusion guidelines are established locally by varying methods within each institution. However, it is prudent that plasma transfusion guidelines be based on the aggregate of local, national, and population-specific evidence. To contribute, we retrospectively evaluated the effect of plasma transfusions on mild elevations of PT-INR often referred to clinically as “mild coagulopathy”.

## Methods

After institutional review board of Baylor College of Medicine approved the study with waiver of informed consent, we conducted a retrospective analysis of transfusion and coagulation-related records from the blood bank and patient electronic medical records (EMR) at Texas Children’s Hospital for the 3-month time period from April to June of 2011. Patient chart review was performed to collect demographic and clinical details. We included all patients who: 1) had a pre-transfusion INR of 1.3 to 1.5, 2) received one or more plasma transfusions, and 3) had a post-transfusion INR checked within 8 h of transfusion. Patients receiving plasma transfusion support during extracorporeal membranous oxygenation (ECMO) or as intraoperative transfusion were excluded from the study. Demographic variables included age, weight, and patient’s location (excluding emergency department and operating rooms, all inpatient hospital units were included). The patient’s clinical description of disease along with the indication for plasma transfusion was extracted from the EMR via blood product orders and provider notes. The results of laboratory coagulation tests performed on the day of plasma transfusion including PT-INR and platelet count were extracted from the same database. Patient hemostatic conditions were categorized into bleeding or non-bleeding as documented by the clinical providers. Next, we assessed any change in the PT-INR, after plasma transfusion. Independent variables included age and body weight. PT was measured using commercial PT reagents on STA-R automatic analyzer (Diagnostica Stago Inc., Parsippany, NJ, USA).

The patient population was characterized into groups based on whether PT-INR decreased after plasma transfusion and whether hemostatic status improved (i.e., bleeding subsided). Fisher’s exact test was used to assess the association between two categorical variables such as post-transfusion decrease in INR (presence or absence), age (<1 vs. ≥1 month), plasma dose (≤10 vs. >10 cc/kg), and bleeding symptoms (bleeding vs. non-bleeding). Association between pre-transfusion PT and bleeding was also assessed. Non-parametric analysis of two related samples employed the Wilcoxon signed rank test to detect significant difference between the pre- and post-transfusion PT-INR values. Mann–Whitney test allowed determination of significant difference in different variables between two categories of patients. In order to determine if an increasing volume of plasma transfused was associated with a change in INR, patients were divided into quartiles based on total plasma volume transfused. From this data, a contingency table (4 × 2) analysis was performed and a Chi-squared (*χ*^2^) *p* value was calculated. *P* values of <0.05 were considered statistically significant.

## Results

A total of 468 plasma transfusions were administered to 285 patients from April 2011 through June 2011. Among these, 60 plasma transfusions (12.8%) were given when PT-INR ≤1.5, ranging from 1.3–1.5. Forty-one patients had post-transfusion PT-INR measurements. Almost half (46.3%) of these plasma transfusions were administered to patients whose PT-INRs were within age-specific normal levels. Of the 41 patients, 39% of patients (*n* = 16) had documented bleeding. Nine patients (22%) were neonates (age ≤30 days) and only five of them had bleeding symptoms. Characteristics of patients (*n* = 41) with plasma transfusion for mildly prolonged PT-INR are listed in Table 1.

**Table 1:** **Characteristics of patients (** ***n*** **= 41) with plasma transfusion for mildly prolonged PT-INR**

**Dependent variable**	**Median**	**IQR**
Age, years	5.00	(0.83–14.00)
Weight, kg	19.00	(8.80–70.80)
Platelet count	121	(73–176)
Volume of plasma transfused, mL	170	(115–333)
Plasma dose, mL/kg	11	(6–15)

*IQR* interquartile range.

There was no statistically significant difference in PT-INR before and after plasma transfusion in the study population (*p* = 0.34) (Table 2). Transfusion of plasma resulted in normalization of PT-INR values in only 15.4% (*n* = 6) of patients with the remainder showing no improvement in PT-INR. Table 3 describes the characteristics of these patients. The median change in PT-INR for patients with normalization was 0.15 (IQR, 0.1–0.2). There was no statistically significant association between PT-INR normalization and higher plasma dose, younger age, or bleeding status (*p* = 0.18, *p* = 0.16, *p* = 0.68), respectively. Increasing plasma transfusion dose was not associated with a change in INR (*p* = 0.78).

**Table 2:** **Coagulation parameters pre- and post-transfusion of plasma for all patients with INR ≤1.5**

**Coagulation test**	**Median (IQR)**	***p*** **value** ^**a**^
Pre-transfusion PT	16.30 (15.30–17.40)	0.34
Post-transfusion PT	16.40 (14.90–17.40)	
Pre-transfusion INR	1.30 (1.20–1.50)	0.42
Post-transfusion INR	1.40 (1.20–1.50)	

*IQR* interquartile range.

^a^Wilcoxon signed rank test.

**Table 3:** **Baseline characteristics of patients with and without normalization of PT-INR**

**Dependent variable**	**No change in PT-INR**	**PT-INR normalized**	***p*** **value** ^**a**^
Age, years	1.25 (0.08–13.0)	7.5 (3.0–14.0)	0.15
Weight, kg	14.9 (7.7–61.6)	44.9 (16.8–70.8)	0.14
Volume of plasma transfused, mL	168 (90–302)	180 (130–250)	0.82
Plasma dose, mL/Kg	12 (6–16)	5 (2–10)	0.06
Platelet count × 10^3^	124 (74–182)	87 (54–120)	0.16

Values are expressed in median (interquartile range).

^a^Mann–Whitney test.

Thirty-nine percent (*n* = 16) of patients were transfused with plasma during a bleeding episode. There was no significant association between pre-transfusion PT and bleeding (*p* = 0.70). Among these patients, the majority (69%, *n* = 11) were clinically deemed as mild: e.g., bleeding around the gums (*n* = 2), slow oozing from an incision site (*n* = 2), a small amount of blood in the endotracheal and nasogastric tube (*n* = 2) and blood-tinged chest-tube output (*n* = 5). The remaining five patients had clinically significant bleeding such as melena (*n* = 2), hemoptysis (*n* = 2), and epistaxis (*n* = 1). Four out of 16 (25%) patients showed improvement in bleeding, and it stopped in 2 out of 4 after local use of anti-fibrinolytics. Of all the bleeding patients, 12% had an improvement in PT-INR values, but were not normalized. Bleeding status was not associated with post-transfusion change in PT-INR (*p* = 1.0). Platelet counts were above 100,000/mL in both bleeding and non-bleeding patients (*p* = 0.74). The volume and dosage of plasma administered to bleeders and non-bleeders were not different (*p* = 0.65, *p* = 0.85) (Table 4).

**Table 4:** **Baseline characteristics of patients with and without bleeding symptoms**

**Variables**	**Bleeding symptoms present**	**Bleeding symptoms absent**	***p*** **value** ^**a**^
Transfusion dose, mL/kg	11 (3–14)	12 (10–17)	0.85
Transfusion volume, mL	203 (90–421)	159 (115–248)	0.65
PT, s	16.3 (15.2–17.6)	16.3 (15.4–17.2)	0.85
Platelet count × 10^3^/mL	116 (74–175)	124 (78–174)	0.74

Values expressed in median (interquartile range).

^a^Mann–Whitney test.

## Discussion

Despite the results of many clinical studies, clinicians still prefer to use plasma as a therapeutic or prophylactic measure to correct mildly prolonged PT-INR based on assumptions of reducing bleeding risk [15]. In our study, we sought to assess the prevalence of plasma use in pediatric inpatients and found it to be high (12.8%). Among our study population, close to half (42%) were less than 1 year old. A majority of these transfusions occurred in the ICU setting including the cardiovascular, neonatal, and pediatric ICUs. This is consistent with a recent large epidemiological study describing the pattern of plasma usage in pediatric hospitals [17]. Our data illustrates the extent of ineffective use of plasma in neonates and in critically ill children, including those with cardiac diseases. Although targeted and therapeutic plasma use in certain clinical conditions can be life-saving (e.g., support of coagulopathy), its general prophylactic benefit in critically ill children is highly doubtful and lacks evidence. In the absence of evidence-based guidelines, pediatric intensivists and physicians in other specialties must make decisions based on presumed probability of benefit to the patient.

In our study, only 39% of patients had bleeding symptoms; the majority (69%) had bleeding events considered mild due to location and very small volume of blood loss. This demonstrates that over half of the patient population with a mild elevation in PT-INR received plasma for non-bleeding causes. Frequent reasons cited in the EMR include pre-procedural prophylaxis and primary volume replacement. The platelet counts were similar in both bleeding and non-bleeding patients indicating that thrombocytopenia was unlikely to have increased concern for overall coagulopathy and therefore lowered the threshold for plasma transfusion. We found no significant association between pre-transfusion PT-INR and bleeding.

Recent evidence-based recommendations for plasma transfusion include: correction of coagulopathy related to DIC, vitamin K, and inherited deficiencies of clotting factors resulting in bleeding [1]. However, our findings and experience suggest that plasma transfusion to attempt correction of an INR <1.5 is a common practice. Although it is not well-documented in the literature, there is a common assumption albeit false that a small increase in INR will reliably predict bleeding risk and that clotting factors in plasma will correct this “derangement” and protect from bleeding. Additionally, there are no pediatric randomized control studies on mild elevation of INR during procedures that offer definite evidence of predictive ability with respect to bleeding. It is also critical, when evaluating hemostasis in neonates, that age-specific normal ranges are determined and referenced. In our study, neonates received 22% (9/41) of transfusions, only half of those with bleeding or potential coagulopathy. Pre-transfusion INR values were all within age-specific normal ranges. It is not surprising that none of these patients had a post-transfusion change in INR. In comparison to adult coagulation times, neonates normally have “more prolonged” coagulation times. The prolongation is usually mild and is not considered pathologic or carrying increased risk of bleeding. Therefore, mildly abnormal coagulation tests without bleeding or strong clinical indicators of coagulopathy should be confirmed abnormal for age and are not an independent indication for plasma therapy in neonates [18].

Our study of pediatric patients [5 (0.14–13.75) median age (IQR) years] examined the effect of plasma transfusion on PT-INR. We found that of the 16% of patients with a post-plasma transfusion change in PT-INR, the change was negligible regardless of increasing dose. We found no significant association between pre-transfusion PT and bleeding. Abdel-Wahab et al. report that plasma corrected INR in only about 16% of adult ICU patients with initial mild elevation in PT-INR in Massachusetts General Hospital [19]. Holland et al. also found insignificant changes in INR results in both pediatric and adult study populations receiving plasma for INR elevations ≤1.5 [20]. Segal et al. systematically reviewed multiple studies and found inadequate proof to determine that abnormal coagulation test results predict bleeding [16]. In a recent review of prophylactic “correction” of INR prior to neurosurgery, the authors concluded that there is no added benefit of plasma transfusion in a non-bleeding patient with mildly prolonged INR [14]. A systematic review published in 2005 identified 25 adult studies, among which none found an association of mildly prolonged coagulation variables with increased risk of bleeding [16]. Thirty percent coagulation factor activity is necessary for maintaining hemostasis. However, Burns et al. demonstrated that prolongation in INR occurs at 50% reduction of multiple coagulation factor activity [21]. Due to assay design and potential interfering clinical factors, PT-INR is an insensitive predictor of low factor levels in critically ill adults, which in part explains their inability to predict bleeding [22].

This study is among very few in the pediatric literature that addresses effectiveness of plasma transfusion for mildly prolonged PT-INR. The results of our study are based on retrospective data, which has an inherent disadvantage in documentation. However, this issue is somewhat mitigated by the bulk of the study being focused on the pre- and post-transfusion delta in INR, which is easily and accurately mined from the laboratory information system or EMR. For a future study with more emphasis on outcomes, we would include a non-transfused patient control group with mildly prolonged coagulation tests for outcome comparison, e.g., peri- or post-procedural bleeding. However, our results signify the effect of plasma transfusion in pediatric patients: plasma transfusion does not significantly improve mildly prolonged PT-INR. This applies to bleeding and non-bleeding patients with the exception of a rapidly bleeding or evolving coagulopathic scenario. In these settings, a mild abnormality may become a serious deficiency during the lag time between specimen collection and reporting of results. Guidelines on the appropriateness of plasma transfusion, as well as relevant test limitations, should be evidence-based when those tests are used as transfusion triggers. Optimal patient care requires pediatric critical care physicians to be aware of low-to-no benefit and potential harm of plasma therapy for mild coagulation abnormalities without supporting indications. Further research, including randomized control trials, is necessary to provide robust evidence for guiding plasma use in children.

## Conclusion

Plasma transfusion in critically ill children does not significantly correct mildly prolonged PT-INR. Pediatric intensivists should avoid prescribing plasma transfusion for an isolated mild increase in PT-INR values with an aim of preventing or controlling bleeding episodes. Guidelines to optimize its use should be formulated based on prospective clinical trials of plasma transfusions with emphasis on transfusion triggers and outcomes related to both bleeding and adverse transfusion reactions.

## Abbreviations

INR: international normalized ratio
PT: prothrombin time
FFP: fresh frozen plasma
ECMO: extracorporeal membrane oxygenator
PICU: pediatric intensive care unit
EMR: electronic medical record
TRALI: transfusion related acute lung injury
DIC: disseminated intravascular coagulation
